# SENJU: a new time-of-flight single-crystal neutron diffractometer at J-PARC

**DOI:** 10.1107/S1600576715022943

**Published:** 2016-02-01

**Authors:** Takashi Ohhara, Ryoji Kiyanagi, Kenichi Oikawa, Koji Kaneko, Takuro Kawasaki, Itaru Tamura, Akiko Nakao, Takayasu Hanashima, Koji Munakata, Taketo Moyoshi, Tetsuya Kuroda, Hiroyuki Kimura, Terutoshi Sakakura, Chang-Hee Lee, Miwako Takahashi, Ken-ichi Ohshima, Tamiko Kiyotani, Yukio Noda, Masatoshi Arai

**Affiliations:** a Neutron Science Section, J-PARC Center, Japan Atomic Energy Agency, 2-4 Shirakata, Tokai, Ibaraki 319-1195, Japan; b Research Center for Neutron Science and Technology, Comprehensive Research Organization for Science and Society, IQBRC Building, 162-1 Shirakata, Tokai, Ibaraki 319-1106, Japan; c Institute of Multidisciplinary Research for Advanced Materials, Tohoku University, 2-1-1 Katahira, Aoba-ku, Sendai, Miyagi 980-8577, Japan; d Neutron Science Division, Korea Atomic Energy Research Institute, 111 Daedeok-Daero 989 Beon-Gil, Yuseong-Gu, Daejeon, Republic of Korea; e Institute of Materials Science, University of Tsukuba, 1-1-1 Tennodai, Tsukuba, Ibaraki 305-8573, Japan; f Department of Pharmacy, Showa Pharmaceutical University, 3-3165 Higashi-Tamagawagakuen, Machida, Tokyo 194-8543, Japan; g European Spallation Source ESS AB, PO Box 176, SE-221 00, Lund, Sweden

**Keywords:** time-of-flight Laue-type single-crystal neutron diffractometer, MLF/J-PARC, sub-millimetre crystals, extreme sample environments

## Abstract

SENJU is a new single-crystal time-of-flight neutron diffractometer installed at BL18 at the Materials and Life Science Experimental Facility of the Japan Accelerator Research Complex (J-PARC). The diffractometer was designed for precise crystal and magnetic structure analyses under multiple extreme sample environments such as low temperature, high pressure and high magnetic field, and for diffraction measurements of small single crystals down to 0.1 mm^3^ in volume. SENJU comprises three choppers, an elliptical shape straight supermirror guide, a vacuum sample chamber and 37 scintillator area detectors. The moderator-to-sample distance is 34.8 m, and the sample-to-detector distance is 800 mm. The wavelength of incident neutrons is 0.4–4.4 Å (first frame). Because short-wavelength neutrons are available and the large solid angle around the sample position is covered by the area detectors, a large reciprocal space can be simultaneously measured. Furthermore, the vacuum sample chamber and collimator have been designed to produce a very low background level. Thus, the measurement of a small single crystal is possible. As sample environment devices, a newly developed cryostat with a two-axis (ω and φ axes) goniometer and some extreme environment devices, *e.g.* a vertical-field magnet, high-temperature furnace and high-pressure cell, are available. The structure analysis of a sub-millimetre size (0.1 mm^3^) single organic crystal, taurine, and a magnetic structure analysis of the antiferromagnetic phase of MnF_2_ have been performed. These results demonstrate that SENJU can be a powerful tool to promote materials science research.

## Abstract 2

SENJU, a time-of-flight Laue-type single-crystal neutron diffractometer, was developed at the Materials and Life Science Experimental Facility of the Japan Accelerator Research Complex (J-PARC). Molecular structure analysis of a sub-millimetre taurine crystal and magnetic structure analysis of an MnF_2_ crystal were performed to evaluate its performance.

## 1. Introduction   

Single-crystal neutron diffraction is one of the most fundamental and powerful techniques to determine the arrangement of light elements and magnetic moments in crystalline materials with high accuracy and reliability. Thus, this technique has been used in various scientific fields, including physics, chemistry, molecular biology, materials science and energy science, and has the potential to be an irreplaceable analytical tool for the development of new functional materials such as proton conductors, hydrogen-absorbing materials and magnets. However, the number of single-crystal neutron diffractometers in the world remains insufficient, and consequently, the number of experiments is limited. Thus, construction of a high-performance versatile single-crystal diffractometer is required to alleviate this limitation and enable the measurement of many important materials.

In designing such a neutron diffractometer, some critical issues must be addressed. One issue concerns the size of the sample and the time required for the measurement. Common belief is that a large single-crystal sample, *e.g.* 5–10 mm^3^ in volume, and a long measurement time, several days to one month, are required for single-crystal neutron diffraction. Thus, single-crystal neutron diffraction is considered as a ‘powerful but limited’ analytical tool. Numerous efforts to overcome these restrictions have been made. One solution involves the combination of a high-flux reactor source and large area detectors such as neutron imaging plates (Niimura *et al.*, 1994 ▸), gas-counting-type ^3^He area detectors (Moon *et al.*, 2007 ▸, 2013 ▸) and CCDs (Ouladdiaf *et al.*, 2006 ▸). BIX-3 (Tanaka *et al.*, 2002 ▸) and BIX-4 (Kurihara *et al.*, 2004 ▸) at JRR-3, BIO-DIFF at FRM-II, and Bio-D (Lee *et al.*, 2013 ▸) at HANARO are monochromator-type diffractometers. LADI (Cipriani *et al.*, 1995 ▸), VIVALDI (Wilkinson *et al.*, 2002 ▸; McIntyre *et al.*, 2006 ▸) and CYCLOPS (Ouladdiaf *et al.*, 2011 ▸) at ILL and KOALA at ANSTO (Edwards, 2011 ▸) are Laue-type diffractometers. Meanwhile, recent progress in the development of accelerator-driven high-intensity spallation neutron sources and time-resolved neutron area detectors have made it possible to achieve highly efficient single-crystal diffraction measurements using the time-of-flight (TOF) Laue technique. SXD (Keen *et al.*, 2006 ▸) at ISIS is equipped with 11 time-resolved scintillator area detectors to cover a large solid angle, which has demonstrated that maximizing the detector coverage greatly reduces the sample size or measurement time. Two recently developed TOF–Laue single-crystal diffractometers, iBIX (Tanaka *et al.*, 2010 ▸; Kusaka *et al.*, 2013 ▸) at J-PARC and TOPAZ (Jogl *et al.*, 2011 ▸) at ORNL, have demonstrated that the combination of a MW-class proton-accelerator-driven spallation neutron source and many area neutron detectors is another solution to mitigate the limitations of single-crystal neutron diffraction.

A further issue involves the sample environments. Low-temperature conditions, below 10 K or sometimes of the millikelvin order, are often required in neutron diffraction experiments on magnetism, superconductive materials and heavy fermion systems to identify magnetic structures that appear only at very low temperature. Although structural studies of molecular crystals may not typically require such low-temperature conditions, the technique may be valuable for certain studies such as those on organic conductors and charge-ordering materials. In general, Bragg reflections in high-*Q* regions become observable at low temperature because of the suppression of the thermal motion of mol­ecules, and consequently highly accurate structure analysis should become possible. The reactivity of the molecule is also suppressed at low temperature; thus, a structural study of the metastable chemical species produced inside the crystalline lattice by photo-irradiation or other external stimuli by the cryo-trapping technique (Kawano *et al.*, 2001 ▸) should also be possible. Other extreme conditions, such as high temperature or pressure or the presence of a magnetic or electric field, are also important for *in situ* studies of crystalline functional materials to clarify the relationship between changes in physical/chemical properties and structures under extreme conditions.

In single-crystal neutron diffraction studies under extreme conditions, the combination of large area detectors and the TOF–Laue technique has a great advantage. This combination allows us to access three-dimensional reciprocal space without moving large ancillary equipment and even with a limited opening angle because of the blockage of ancillary equipment. Furthermore, unpredictable features, such as diffuse scattering or satellite reflections derived from structural changes, can be easily detected using this combination. Previous studies using the TOF–Laue single-crystal diffractometer FOX at KENS have clearly demonstrated that the TOF–Laue technique is effective in providing an overview of the intensity distribution of both Bragg reflections and weak scattering such as diffuse scattering and satellite reflections at low temperature and/or in a magnetic field (Takahashi *et al.*, 2007 ▸).

For these reasons, a new TOF–Laue single-crystal neutron diffractometer, SENJU, was built at the Materials and Life Science Experimental Facility (MLF) of the Japan Proton Accelerator Research Complex (J-PARC), one of the brightest spallation neutron sources in the world. SENJU, named after the ‘thousand-armed’ Goddess of Mercy, was made mainly for physics, chemistry and materials science. The maximum cell length of sample crystals and minimum *d* value were chosen as 50 and 0.4 Å, respectively (Tamura *et al.*, 2012 ▸). These values were selected for the study of the superstructure of organic conductors or large supramolecular systems. Large area detectors were adopted to achieve efficient measurements of three-dimensional reciprocal space with white neutrons. To expand the capability of measurable crystals and possible measurement conditions, SENJU was designed to perform neutron structure analyses of inorganic and organic single crystals with volumes of 0.1 mm^3^ within a realistic beam time and to accept various types of extreme condition devices such as a cryostat, superconducting magnet or high-temperature furnace. Another single-crystal diffractometer, iBIX, is used for protein crystallography at J-PARC; thus, SENJU and iBIX are complementary diffractometers that cover most scientific fields related to crystallography.

After two years of construction, SENJU received the first neutron beam on 5 March 2012. After the primary conditionings (Oikawa *et al.*, 2014 ▸), various diffraction measurements were performed to evaluate the performance of SENJU. This paper describes the design of SENJU and presents the results of some typical measurements.

## 2. Instrument design of SENJU   

### 2.1. Neutron optics   

**Figure 1:**
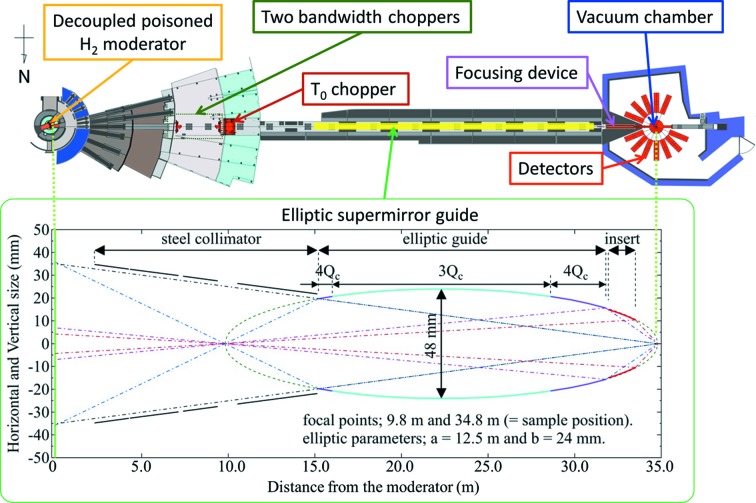
Design of the neutron optics for SENJU.

**Figure 2:**
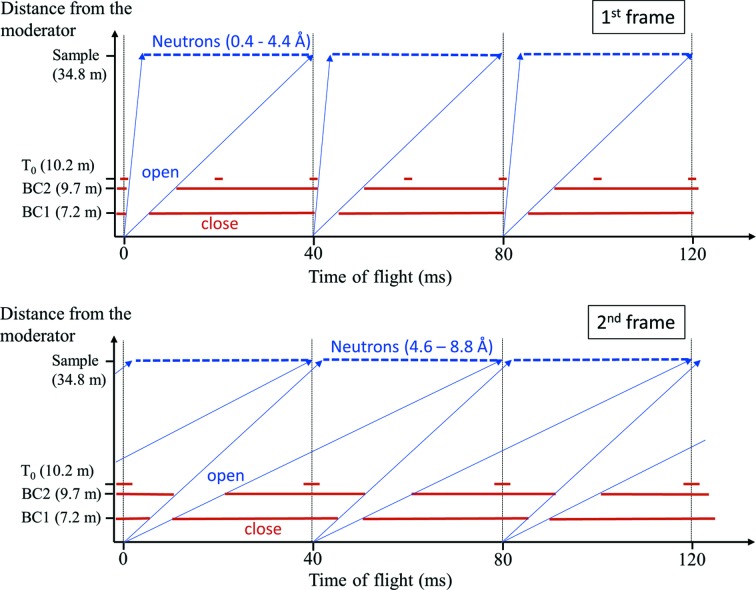
Schematic diagram of the neutron paths for the different choices of incident wavelength. BC1 and BC2 represent the bandwidth choppers at *L* = 7.2 m (BC1) and 9.7 m (BC2). (Top) First frame. The T_0_ chopper works at 50 Hz so as not to cut the short-wavelength neutrons. (Bottom) Second frame. The T_0_ chopper works at 25 Hz so as not to cut the available wavelength of neutrons.

SENJU is situated at beamline BL18 of MLF, a 25 Hz target station at J-PARC. SENJU views a 20 K poisoned *para*-hydrogen decoupled moderator to prevent the broadening of Bragg peaks and makes it possible to observe weak satellite reflections or diffuse scattering around main Bragg peaks. The primary flight path (*L*_1_; moderator-to-sample distance) of SENJU is 34.8 m. Fig. 1 ▸ presents a schematic view of the neutron optics. One T_0_ chopper at *L* = 10.2 m blocks the high-energy neutrons and γ-rays from the neutron source, and two bandwidth choppers at *L* = 7.2 and 9.7 m extract the required neutron wavelength. Here, *L* stands for the distance from the moderator. The available wavelength of the incident neutrons is 0.4–4.4 Å (first frame), 4.6–8.8 Å (second frame) and 9.0–13.2 Å (third frame). Fig. 2 ▸ presents a schematic diagram of the neutron paths for the different choices of incident wavelength. An elliptical supermirror neutron guide was designed to focus the incident neutron beam and enhance the intensity at the sample position. The supermirror guide is set at *L* = 15.2–31.8 m, and an additional 1.56 m focusing supermirror device on a movable stage is positioned at the end of the guide. Without the additional focusing device (called the high-resolution mode), the beam divergence and available neutron flux at the sample position with 1 MW accelerator power are 0.6° and 0.6 × 10^6^ n s^−1^ mm^−2^, respectively. With the focusing device (called high-intensity mode), the divergence and flux are 0.9° and 1.3 × 10^6^ n s^−1^ mm^−2^, respectively.

### 2.2. Sample chamber and detectors   

**Figure 3:**
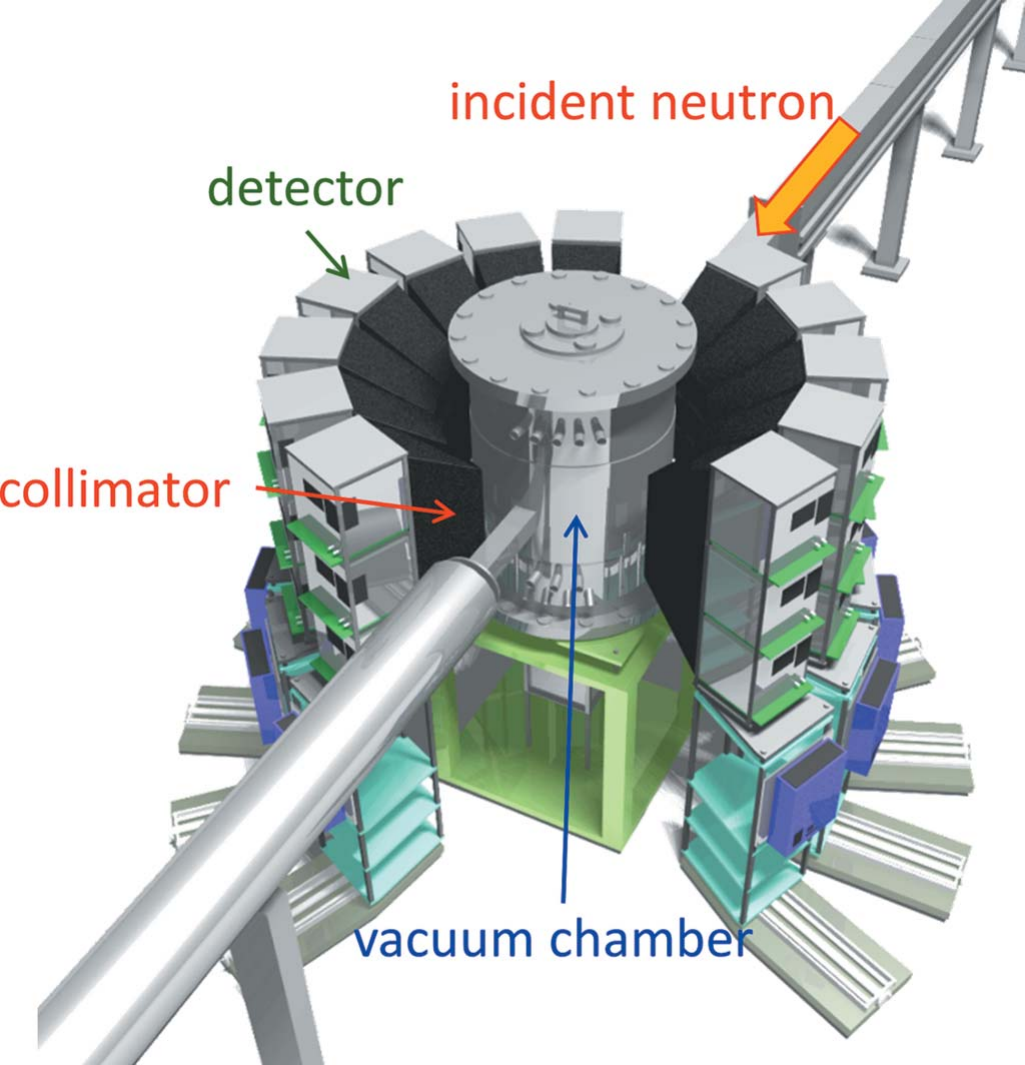
Schematic view of the SENJU main unit. One detector is just visible beneath the sample vacuum chamber.

Fig. 3 ▸ presents a schematic view of SENJU. SENJU has a top-loading vacuum sample chamber and 37 two-dimensional wavelength-shifting-fiber (WLSF)-type scintillator detectors (Kawasaki *et al.*, 2014 ▸).

The vacuum sample chamber, with a 800 mm diameter, has ϕ400 mm and ϕ800 mm diameter flanges at the top of the chamber. The specifications of these flanges are in accordance with the MLF standard; hence, many MLF common-use sample environment devices such as a furnace or dilution cryostat can be mounted in the sample chamber. The sample chamber has additional vacuum tubes at both the upstream and downstream sides along the direct beam to reduce air scattering of the incident and transmitted neutrons. A collimator comprising boron nitride and boron carbide in the vacuum tube at the upstream side blocks off the scattered neutrons at the aluminium window of the sample chamber. The sample chamber can be easily removed.

One area detector has a sensitive area of 256 × 256 mm, containing 64 × 64 pixels (one pixel is 4 × 4 mm in size) surrounded by an outer frame with a width of 22 mm. One detector array module is composed of vertically stacked three-detector segments, and 12 detector modules (accommodating 36 detectors) are arranged cylindrically around the chamber. An additional detector is positioned underneath the chamber. The distance from the sample position to the detector on the equatorial plane (*L*_2_) is 800 mm. One detector covers 0.103 steradians, and in total 30.2% of 4π steradians is covered by the area detectors. The minimum and maximum 2θ covered by the detectors in the horizontal direction are 13.0 and 167.0°, respectively. One detector array module also covers ±30° in the vertical direction. Each area detector has a square-frustum-shape collimator made of B_4_C resin between the detector and the sample chamber to block off the neutrons scattered from the beamline components such as the slit, aluminium windows of the sample chamber and beam dump. The combination of the vacuum sample chamber and collimators inside and outside of the chamber results in a low-background diffraction measurement at SENJU.

### 2.3. Sample alignment and sample environment devices   

#### 2.3.1. Sample alignment at SENJU   

**Figure 4:**
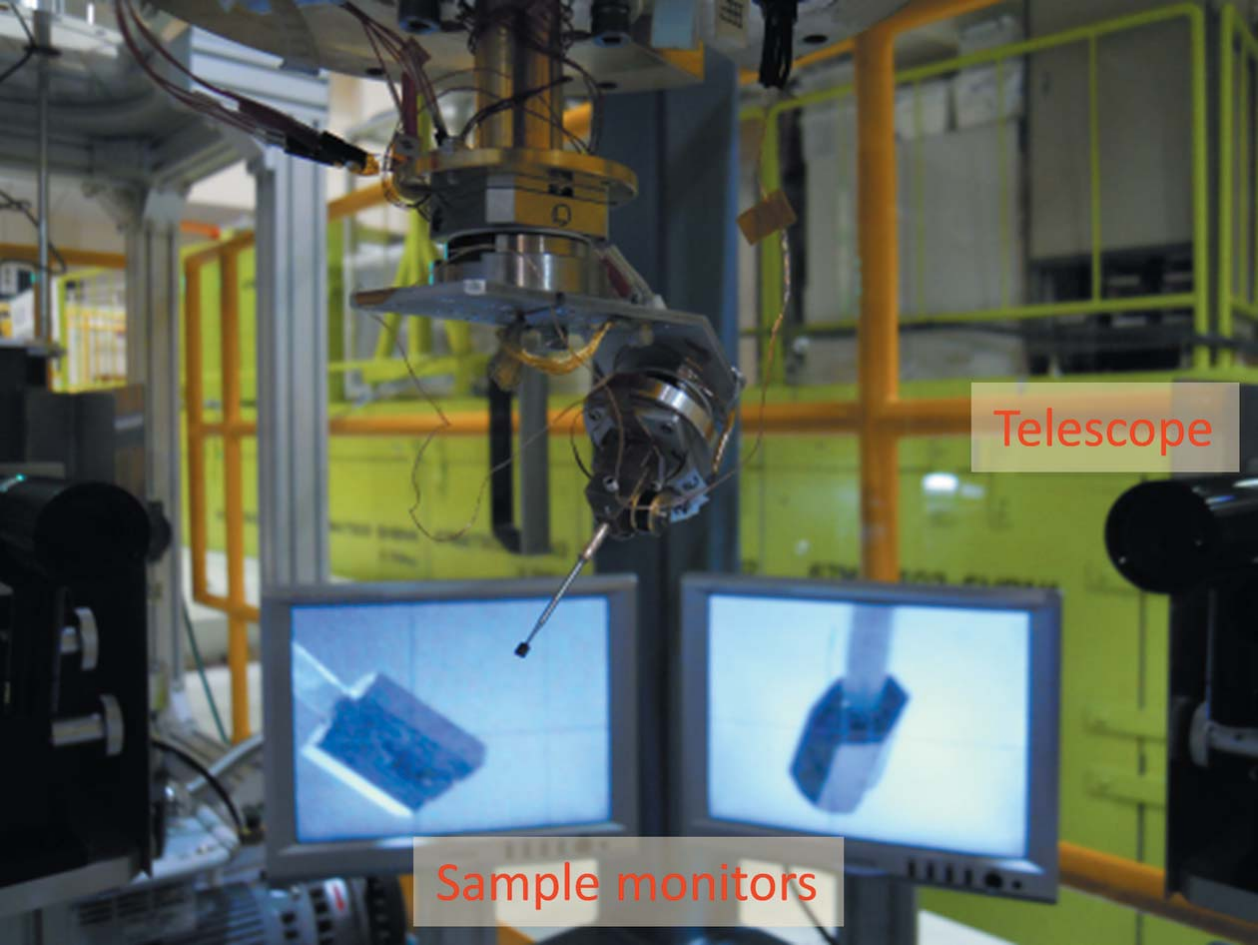
Off-line sample alignment stand for SENJU. The stand has two telescopes and monitors to adjust the position of the sample crystal.

In the standard diffraction measurement at SENJU, a sample crystal is mounted on a sample environment device, and the sample position is adjusted on a specially designed sample alignment stand. As shown in Fig. 4 ▸, the sample alignment stand comprises the ϕ400 mm MLF standard flange and two CCD cameras to observe the sample position. After the sample alignment, the device with the sample is moved into the sample chamber. To perform diffraction measurements under various types of extreme conditions, several sample environment devices have been developed and commissioned for SENJU, as described below.

#### 2.3.2. Sample stick with ω- and φ-axis piezo-rotating goniometer   

SENJU is based on the TOF–Laue technique, and consequently, a large reciprocal space can be scanned with one crystal orientation. However, the orientation of the sample crystal must be changed several times during the experiment to scan the entire crystallographically independent reciprocal space. To cover all of the independent reciprocal space of a low-symmetry crystal, at least two rotational axes are required. In a typical diffraction experiment with a four-circle single-crystal neutron diffractometer, a sample crystal and sample environment device (*e.g.* a cryostat) are mounted on a large goniometer with χ, φ and ω axes to rotate the sample crystal to access arbitrary points in the reciprocal space. However, SENJU has a large vacuum chamber at the sample position, and there is no space to place a large goniometer. Therefore, a sample stick with a ϕ400 mm MLF standard flange and a compact two-axis goniometer system with rotatable ω and φ axes and fixed χ axis was developed for ambient-temperature diffraction measurements. As the rotational devices, piezo-rotating motors (attocube systems AG, ANR101/RES for the ω axis, and ANR50/RES for the φ axis) were adopted because the rotational devices must work under vacuum and extreme conditions. The goniometer has an XY stage on the φ axis. The XY stage does not shade the detectors. A sample crystal is usually mounted on an aluminium or vanadium pin and then mounted on the XY stage. In the standard diffraction measurement for structure analysis at SENJU, the sample crystal is rotated approximately 5–15 times (depending on the crystal symmetry and required *Q*_max_ value) by the ω- and φ-axis goniometer to measure as many Bragg reflections as possible spread over the three-dimensional reciprocal space.

#### 2.3.3. Cryostat with ω- and φ-axis piezo-rotating goniometer   

Low temperature, below 10 K, is one of the most important measurement conditions for neutron diffraction measurements in physics and materials science, and a cryostat is frequently used in single-crystal neutron diffraction experiments. Hence, a versatile cryostat for SENJU was developed as a first priority.

**Figure 5:**
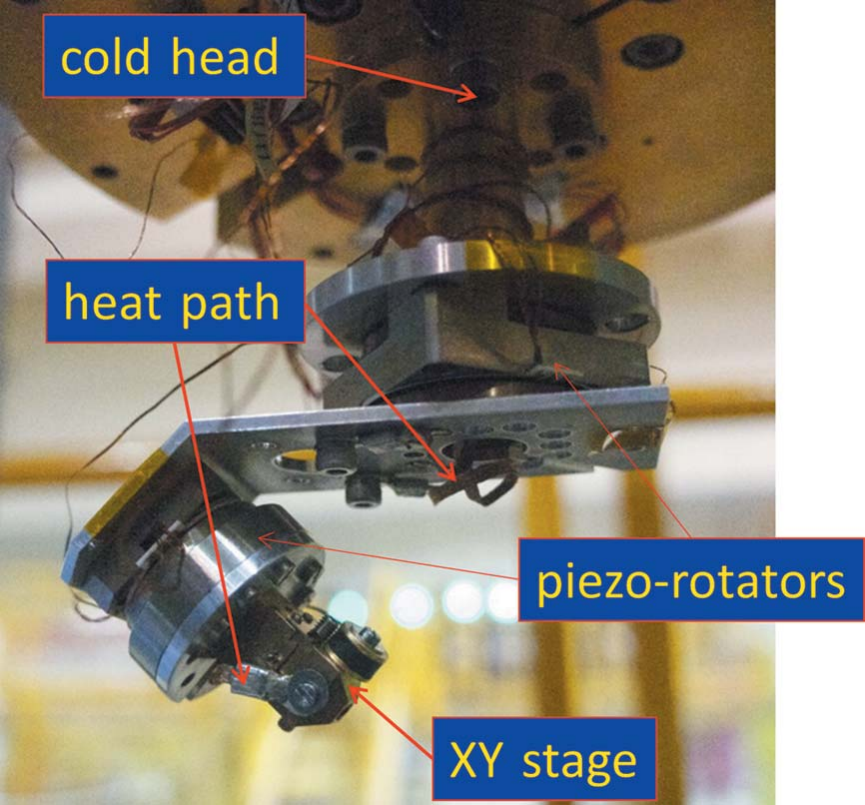
Cold head and the ω- and φ-axis goniometer of the cryostat for low-temperature diffraction measurement at SENJU.

The cryostat for low-temperature measurements comprises the ϕ400 mm MLF standard flange, a 4 K GM-type two-stage refrigerator (Iwatani Industrial Gases Co., HE05), and an ω- and φ-axis goniometer at the cold head of the refrigerator. The goniometer is similar to that for ambient conditions; however, the piezo-rotating motors used in this device are higher-torque types (attocube systems AG, ANR240/RES for the ω axis, and ANRv220/RES for the φ axis) because the torque of the piezo-device decreases at low temperature. The cold head of the refrigerator and the XY stage of the goniometer are connected with copper wires as heat paths. The procedure to adjust the sample position is the same as that for the ambient-condition sample stage. Fig. 5 ▸ shows the cold head and ω- and φ-axis goniometer of the cryostat. The minimum temperature at the sample position is approximately 3.5 K, and it takes approximately 4.5 h to cool from room temperature to the minimum temperature. Once the sample reaches a target temperature, the remainder of the procedure for the diffraction measurement is the same as that for the ambient-temperature measurement.

#### 2.3.4. Other sample environment devices   

Currently, a vertical-field superconducting magnet with ω axis (maximum magnetic field and minimum temperature of 7 T and 50 mK, respectively) and a niobium furnace with ω axis (maximum temperature of 1873 K) are available at SENJU. A 2 K cryostat and dilution insert (minimum temperature of 50 mK) and a bottom-loading-type cryogen-free ^3^He refrigerator are under construction.

Other sample environment devices optimized for SENJU have also been developed. A piston-cylinder-type high-pressure cell (maximum pressure of 2 GPa), a cryostat for *in situ* low-temperature Xe light exposure diffraction measurement and a device for *in situ* electric field diffraction measurement have been commissioned.

### 2.4. Data processing and visualization software   

**Figure 6:**
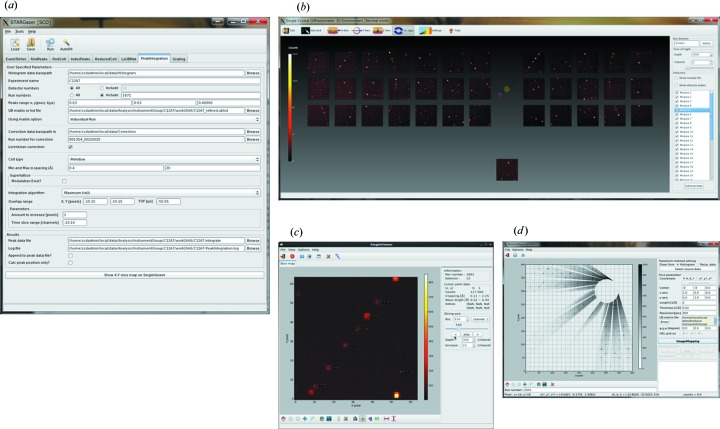
Screenshots of *STARGazer for SENJU*. (*a*) Graphical user interface of the data processing component. (*b*) Data viewer for all 37 detectors. The circle between the two banks of detectors corresponds to the incident neutron beam. (*c*) Data viewer and analyzer for each detector. (*d*) Data viewer in reciprocal space.

Software for data processing, *i.e.* making a reflection (HKLF) file from raw data, and data visualization is one of the essential components for a single-crystal diffractometer. For SENJU, the program *STARGazer for SENJU* is used for the data processing and visualization. *STARGazer for SENJU* is a modification of the program *STARGazer* (Ohhara *et al.*, 2009 ▸) developed for the iBIX single-crystal diffractometer at MLF/J-PARC. *STARGazer for SENJU* can execute data processing, visualization of measured diffraction patterns on all 37 detectors simultaneously, detailed visualization of the data on each detector and visualization of reciprocal space. The data processing includes data conversion from the raw event data into histogram-format data, peak search from the histograms, indexing, refinement of the UB matrix, intensity correction of the histogram-format data by normalization with incoherent neutron scattering data of a vanadium–nickel alloy (null-alloy) in which Lambert’s cosine law was applied, and integration of Bragg reflections. The normalization with the null-alloy data includes intensity corrections of the wavelength dependence of incident neutrons and the position dependence of the detector efficiency. The generated HKLF file can be seamlessly fed into the structure analysis program *Jana2006*, thanks to the authors of the program (Petricek *et al.*, 2014 ▸). Fig. 6 ▸ presents screenshots of *STARGazer for SENJU*.

## 3. Examples of measurements   

### 3.1. Diffraction measurement of a sub-millimetre size single crystal: structure analysis of a 0.1 mm^3^ size taurine single crystal   

**Figure 7:**
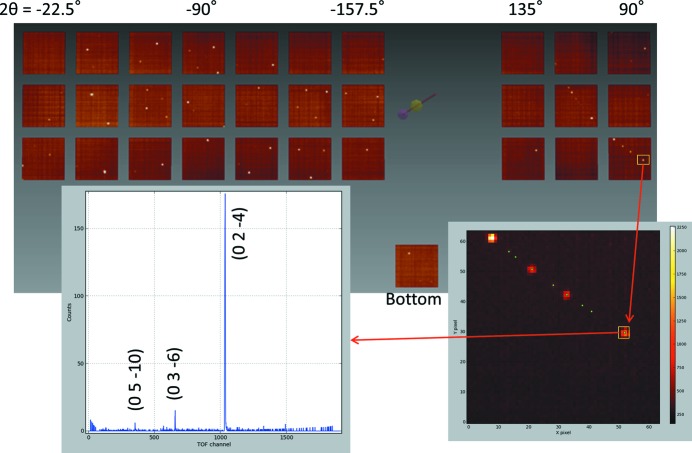
TOF–Laue diffraction image of a taurine single crystal with 0.1 mm^3^ volume measured at SENJU. The numbers at the top edge are the 2θ values of the center of each detector. The two-dimensional graph at the lower left presents the TOF profile in the yellow rectangle of the TOF–Laue diffraction image.

**Figure 8:**
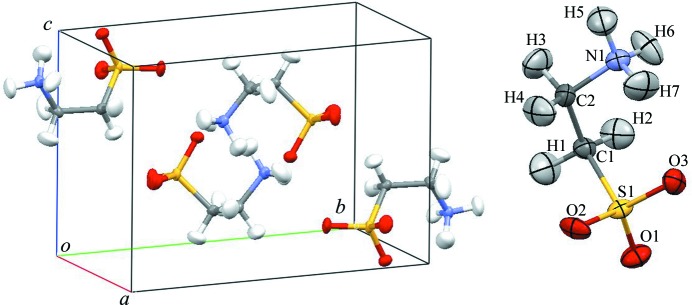
Crystal packing and molecular structure of taurine determined by single-crystal neutron structure analysis with a 0.1 mm^3^ volume single crystal at SENJU. Displacement ellipsoids are shown at the 50% probability level.

One of the most important aims of SENJU is diffraction measurements of a sub-millimetre size single crystal within a realistic beam time. To study the feasibility of such a measurement, structure analysis of a small single crystal was performed. A spherical single crystal of taurine (monoclinic, *a* = 5.27, *b* = 11.68, *c* = 7.93 Å, β = 93.82°) with a volume of 0.1 mm^3^ was selected as the test sample. The sample was fixed on a glass rod with Araldite glue and mounted on the sample stick with the ω- and φ-axis goniometer for ambient-temperature measurement. In this measurement, two detector modules (six detectors) were not yet installed and 31 detectors were available. The measurement was performed at room temperature, and the accelerator power was 260 kW. TOF diffraction data were collected with six crystal orientations, and the neutron exposure time was approximately 30 h for one orientation. The total exposure time was 7.5 d. Fig. 7 ▸ presents the TOF diffraction image of the taurine single crystal. The collected data were processed with *STARGazer for SENJU*, as described in §[Sec sec2.4]2.4. Overall, 980 reflections [*I* > 4σ(*I*)] were observed, and the minimum *d* value was 0.5 Å. The structure refinement was performed using *Jana2006*. The atomic positions and anisotropic displacement parameters of all the H and non-H atoms were refined without any restraints. Fig. 8 ▸ presents an elliptical atomic model of the obtained molecular structure and crystal packing. The number of refined parameters was 129, and all the refined parameters agreed well with the reported values determined by single-crystal neutron diffraction (Briant & Jones, 1997 ▸), as shown in Table S1 of the supporting information. The final *R* value was 7.14% [*I* > 4σ(*I*)] and the crystallographic and statistical data are given in Table S2. Although the required beam time for neutron structure analysis depends not only on the crystal size but also on the cell volume, space group, crystallinity, beam power and other factors of the sample crystal, this result suggests that neutron structure analysis of a 0.1 mm^3^ size single crystal will be practicable within a realistic beam time (one day to one week), especially after an accelerator power of 1 MW is achieved at J-PARC in the near future.

### 3.2. Magnetic structure analysis of a manganese fluoride single crystal at low temperature with the cryostat with ω- and φ-axis goniometer   

**Figure 9:**
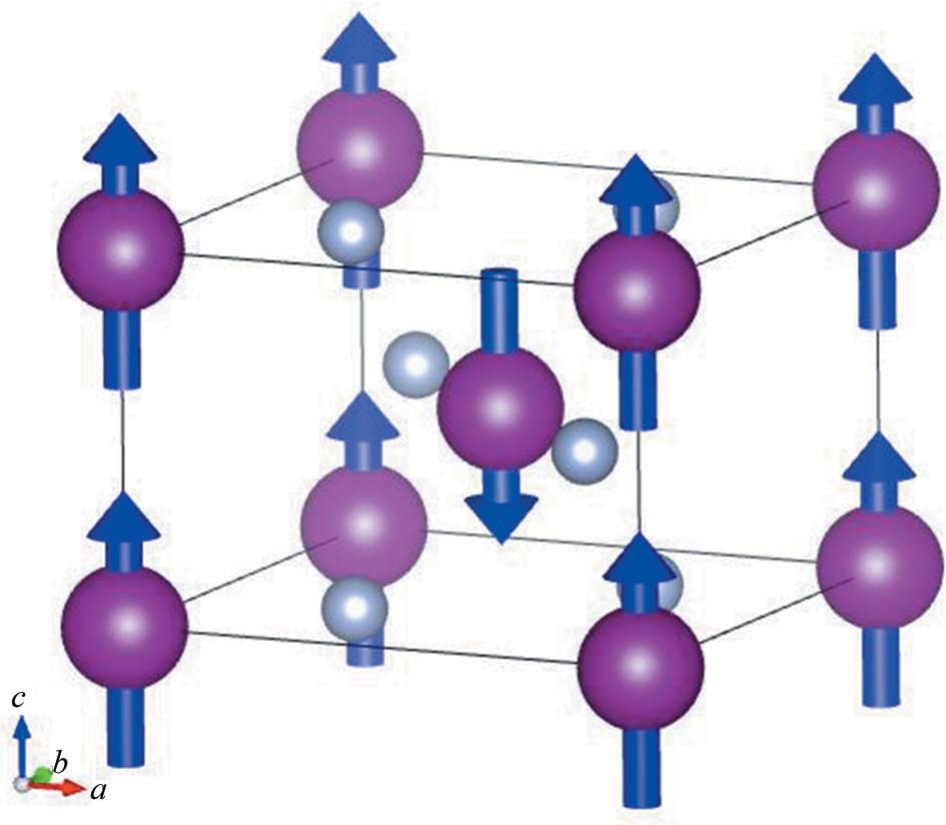
Reported crystal and magnetic structure of MnF_2_ in the antiferromagnetic phase. The small gray spheres represent F^−^, the large purple spheres represent Mn^2+^ and the blue arrows indicate the direction of the magnetic spin of Mn^2+^.

**Figure 10:**
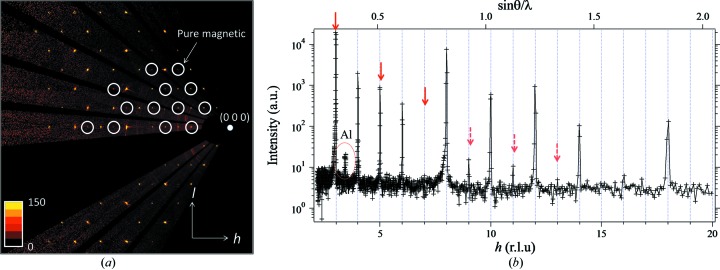
(*a*) (*h*0*l*) plane of the diffraction from MnF_2_ at 4.3 K. The white circles indicate pure magnetic reflections. (*b*) Profile of the [*h*00] axis. The red arrows indicate pure magnetic reflections. As described in the main text, some pure magnetic reflections in the high-*Q* region (dashed arrows) may be heavily affected by the multiple scattering effect. Al labels the strongest of the weak parasitic powder lines from scattering from the cryostat heat shields.

As described in §[Sec sec2.3.3]2.3.3, low temperature is one of the most important and frequently used measurement conditions at SENJU, and we developed a cryostat with an ω- and φ-axis goniometer. As an example of low-temperature measurement and to verify the performance of the cryostat, magnetic structure analysis of a manganese fluoride (MnF_2_) single crystal was performed. The measurement also aimed to examine the capability of SENJU for magnetic structure analyses. MnF_2_ has a tetragonal crystal structure (*P*4_2_/*mmm*) with the cell parameters *a* = *b* = 4.874, *c* = 3.299 Å and has a paramagnetic–antiferromagnetic phase transition at 75 K (Erickson, 1953 ▸) with no structural change. In the antiferromagnetic phase, the magnetic moments of the neighboring Mn^2+^ ions in the body diagonal direction are antiparallel along the *c* axis. The reported crystal and magnetic structure of MnF_2_ is shown in Fig. 9 ▸. A 2 × 2 × 2 mm MnF_2_ single crystal was fixed on a vanadium rod and mounted on the XY stage of the cryostat with the ω- and φ-axis goniometer. After the cryostat had been placed in the sample vacuum chamber, the temperature of the cold head was set to 4.0 K. The final temperature at the XY stage of the goniometer, the closest temperature sensor to the sample, was 4.3 K. In the diffraction measurement, the neutron exposure time was 10 h. Fig. 10 ▸(*a*) presents an observed diffraction image of the reciprocal space in the (*h*0*l*) plane. Many pure magnetic reflections of the antiferromagnetic phase (white circles) were observed. A few powder rings from the radiation shields of the cryostat are observed, but these rings are very weak compared with the Bragg peaks from the sample crystal. In this measurement, 2084 reflections including 80 pure magnetic reflections [*I* > 3σ(*I*)] were observed. Fig. 10 ▸(*b*) presents a profile of the [*h*00] axis. A preliminary structure refinement suggested that the magnetic moment of Mn^2+^ and the extinction correction parameter were strongly correlated with each other, and some pure magnetic reflections in the high-*Q* region (sinθ/λ > 0.8) may be heavily affected by the multiple scattering effect. Thus, the magnetic moment of Mn^2+^ was fixed at the nominal value, μ(Mn^2+^) = 5 µ_B_, and 1785 reflections including 25 pure magnetic reflections in the sinθ/λ < 0.8 region were used in the refinement. The final *R* value was 4.60% [*I* > 3σ(*I*)]. The structural parameters and the crystallographic and statistical data are shown in Tables S3 and S4, respectively. This result indicates that the cryostat has sufficient cooling ability and that the neutron scattering from the cryostat rarely disturbs the structure analysis.

## 4. Conclusion   

A new TOF–Laue-type single-crystal neutron diffractometer, SENJU, has been launched at J-PARC. SENJU contains 37 scintillator area detectors and a vacuum sample chamber with an MLF standard flange that can accept various types of sample environment device. A newly developed cryostat with a piezo-rotating goniometer allows ω- and φ-axis rotation of a sample crystal at low temperature (>3.5 K). Measurement of a taurine crystal demonstrated that diffraction measurements of organic single crystals with volumes of 0.1 mm^3^ should be practicable within a realistic beam time at SENJU, particularly after the accelerator achieves 1 MW operation. Measurement at 4 K demonstrated that the cryostat has sufficient cooling ability and that the powder pattern from the cryostat rarely disturbed the structure analysis. These results indicate that the main purpose of SENJU, crystal and magnetic structure analyses of inorganic/organic crystalline materials under extreme conditions, has been achieved. The general user program of SENJU has already started and the devices described in this paper are available for this program.

## Supplementary Material

The supporting information includes four tables. Structural parameters of taurine, crystallographic and statistical data of taurine, structural parameters of MnF2, and crystallographic and statistical data of MnF2.. DOI: 10.1107/S1600576715022943/fs5124sup1.pdf
