# Supplementary material for: SENJU: a new time-of-flight single-crystal neutron diffractometer at J-PARC
Source: J Appl Crystallogr. 2016 Feb 1;49(Pt 1):120–7. doi: 10.1107/S1600576715022943 (PMC4762571; doi:10.1107/S1600576715022943)
Supplement: Supplementary file 1 [file j-49-00120-sup1.pdf]

Supporting information

Table S1 Atomic positional parameters (e.s.d. in parentheses) and isotropic thermal parameters of taurine. (upper column) The ϕ0.5 mm taurine crystal measured by SENJU. (lower column) Parameters in the reference determined by single-crystal neutron diffraction (Briant & Jones, 1997).

Atom	*x*	*y*	*z*	U_{iso}
S1	0.2037(11)	0.3481(6)	0.3522(6)	0.020(2)
	0.2047(14)	0.3498(7)	0.3523(14)	0.0282
O1	0.3404(7)	0.2414(3)	0.3541(4)	0.0343(13)
	0.3399(8)	0.2412(4)	0.3534(8)	0.0400
O2	-0.0631(7)	0.3372(4)	0.2924(5)	0.0337(12)
	-0.0644(8)	0.3369(4)	0.2937(7)	0.0391
O3	0.2326(7)	0.4108(4)	0.5114(4)	0.0296(12)
	0.2330(7)	0.4111(4)	0.5121(7)	0.0349
C1	0.3398(6)	0.4391(3)	0.1997(3)	0.0242(9)
	0.3403(7)	0.4383(4)	0.1987(8)	0.0323
H1	0.3188(16)	0.3914(6)	0.0805(7)	0.050(3)
	0.3227(19)	0.3920(10)	0.0831(20)	0.0616
H2	0.5434(12)	0.4458(7)	0.2378(8)	0.045(2)
	0.5417(14)	0.4457(7)	0.2409(13)	0.0477
C2	0.2100(6)	0.5553(3)	0.1815(3)	0.0261(9)
	0.2116(7)	0.5556(3)	0.1822(5)	0.0290
H3	0.2804(17)	0.6007(6)	0.0752(8)	0.054(3)
	0.2823(20)	0.5997(8)	0.0744(18)	0.0635
H4	0.0037(12)	0.5469(7)	0.1639(9)	0.056(3)
	0.0044(14)	0.5461(7)	0.1635(14)	0.0637
N1	0.2639(4)	0.6294(2)	0.3312(2)	0.0247(7)
	0.2641(7)	0.6296(3)	0.3315(5)	0.0310
H5	0.1978(14)	0.7098(7)	0.3040(8)	0.046(2)
	0.1913(20)	0.7099(11)	0.3056(19)	0.0517
H6	0.4537(14)	0.6346(7)	0.3643(9)	0.050(3)
	0.4503(24)	0.6366(10)	0.3625(19)	0.0473
H7	0.1729(16)	0.5987(6)	0.4321(8)	0.055(3)
	0.1764(28)	0.5971(12)	0.4336(25)	0.0721

U_{iso} for the atoms of taurine is the 'equivalent' isotropic temperature factor: $U_{iso} = (U_1.U_2.U_3)^{1/3}$ where U_1, U_2 and U_3 are the principal axes of the thermal ellipsoids.

Table S2 Crystallographic and refinement data of taurine.

Formula	$C_2H_7NO_3S$
Formula Weight [g mol^{-1}]	125.14
Crystal size	ϕ0.5 mm (sphere shape)
Crystal system	Monoclinic
Space group	$P2_1/c$
λ [Å]	0.6-4.4
a [Å]	5.2717(1)
b [Å]	11.6787(1)
c [Å]	7.9328(1)
β [°]	93.824(1)
V [Å^3]	487.308(7)
Z	4
F(000)	66.94
T [K]	293
No. of total reflections	2958
No. of total reflections (I > 4σ(I))	980
Complenteness	0.62 (*d* > 0.5 Å)
R_{int}	N/A*
R(F), Rw(F) (I > 4σ(I))	7.16, 7.49

*Each reflection collected in different settings was collected at different wavelength and scaling factors for each orientation, and extinction were corrected before calculation of R_{int}. Consequently, R_{int} was model dependent so we do not describe it here.

Table S3 Atomic positional parameters (e.s.d. in parentheses) and anisotropic thermal parameters of MnF_2.

Atom	x	y	z	Magnetic moment[μ_b]
	U_{11}[Å]	U_{33}	U_{12}	
Mn	0	0	0	5(fixed)
	0.00165(5)	0.00152(8)	-0.00022 (5)	
F	0.30459(3)	0.30459(3)	0	
	0.00418(4)	0.00354(7)	-0.00174(3)	

Table S4 Crystallographic and refinement data of MnF_2.

Formula	MnF_2
Formula Weight [g mol^{-1}]	92.9349
Crystal size	2 × 2 × 2 mm
Crystal system	Tetragonal
Space group	$P4_2/mnm$
λ [Å]	0.6-4.4
a [Å]	4.874(2)
c [Å]	3.299(1)
V [Å^3]	78.36(6)
Z	2
F(000)	15.156
T [K]	4.0
No. of total reflections	1867 (pure magnetic: 25, pure nuclear: 959, mix: 883)
No. of total reflections (I > 3σ(I))	1785 (pure magnetic: 25, pure nuclear: 929, mix: 831)
Complenteness	0.74(d > 0.625 Å)
R_{int}	N/A*
R(F), Rw(F) (I > 3σ(I))	4.68, 6.64

*Each reflection collected in different settings was collected at different wavelength and scaling factors for each orientation and extinction were corrected before calculation of R_{int}. Consequently, R_{int} was model dependent so we do not describe it here.
